# Announcing the Launch of *CHEST Pulmonary* and the *CHEST* Portfolio of Journals

**DOI:** 10.1016/j.chpulm.2023.100001

**Published:** 2023-02-05

**Authors:** Matthew C. Miles

**Affiliations:** Atrium Health Wake Forest Baptist Medical Center and Wake Forest School of Medicine, Winston-Salem, NC

The year 2023 marks the launch of the *CHEST* portfolio of journals. As expansions to the long-standing flagship journal *CHEST,* we welcome two new open-access journals, *CHEST Critical Care**^1^* and *CHEST Pulmonary*. Why begin new open-access journals in the field of critical care, pulmonary, and sleep medicine? Two primary factors have led to this opportunity: the accelerating volume of high-quality research manuscripts submitted to *CHEST* and the move toward open science publishing models. For more information on our decision to expand, see the accompanying editorial in *CHEST.*^2^

## A Proud Heritage From *CHEST*

*CHEST Pulmonary* inherits a unique legacy. In 1935, Murray Kornfeld, a patient, hosted the first meeting of what would become the American College of CHEST Physicans.^3^ That same year, the first issue of *Diseases of the Chest* appeared, which was the predecessor of the journal *CHEST*. Over subsequent decades, the College’s dedication to high-quality professional education in diseases of the chest has led to immeasurable positive impact through research publications, live learning courses, the CHEST annual meeting, and many other activities. Underpinning all these activities is the patient-centered energy and curiosity that characterized Mr Kornfeld’s vision. Thus, the vision of *CHEST Pulmonary* is to be a respected source of clinically relevant research and patient management guidance for pulmonary and sleep medicine teams worldwide.

## The Scope of *CHEST Pulmonary*

Authors and readers already see *CHEST* as a trusted source of clinically relevant research. Expanding the *CHEST* portfolio provides authors with even greater opportunity to reach our portfolio’s broad audience. In addition, certain areas of pulmonary and sleep medicine research are not targeted by the leading journals in our space. These include implementation science, medical decision-making, artificial intelligence applications, quality improvement, and education research among others. Important publications related to clinical research (such as early-phase clinical trials, descriptions of study designs, health care and public policy reports, and articles that support new guideline releases) will also find a home in *CHEST Pulmonary*. At the risk of stating the obvious, *CHEST Pulmonary* is seeking high-quality manuscripts in all clinically relevant areas of pulmonary and sleep medicine research.

## Open Science Has a Home in *CHEST Pulmonary*

Over the last decade, there has been a growing consensus that open access to research data and results is the desired future state, a scenario often termed “open science.” Several funding agencies and international organizations have issued statements that support and, in some cases, mandate this future.^4^, ^5^, ^6^, ^7^ Although these statements differ from one another and mandates for open data-sharing will require complex solutions, one can see that the international era of open science has now arrived. As a gold open access journal, *CHEST Pulmonary* is positioned as an acceptable target journal under all these directives, and our goal is to get important findings into the hands of as many readers as possible.

## Publishing in *CHEST Pulmonary*

Just as with the journal *CHEST*, *CHEST Pulmonary* accepts direct submissions, does not require any specific formatting at original submission, and uses a double-blind peer review process. Authors whose work is reviewed favorably by *CHEST*, but is not accepted, can receive an offer for easy transfer of their submission (including its reviews) for consideration in *CHEST Pulmonary*. In all cases, manuscript decisions will be made rapidly, providing authors with prompt feedback to move their work forward.

Instead of subscription fees, open access models collect article processing charges from authors after manuscript acceptance. The *CHEST* editorial team is committed to ongoing attention to this arrangement. There are no fees required for article submission or review, and decisions regarding manuscripts remain separate from payment considerations. In addition, fees may be reduced or eliminated in some circumstances, which include institutional open access agreements^8^ and the Research4Life partnership benefiting investigators in lower income countries.^9^ As a society journal, members of CHEST will also receive a discount on article processing charges. Our portfolio’s marketing team will continue to increase the visibility of authors’ work, including leveraging web and multimedia content plus CHEST social media to heighten the impact of important findings.

## With Gratitude

The launch of a new journal requires the efforts of many people, a few very publicly and most behind the scenes. To this end, I have several people to thank for their work prior to our Journal launch and in anticipation of the work ahead. Our editorial board structure includes eight content area teams, each of which is led by an Associate Editor who has recruited his or her editorial board to represent a breadth of expertise as they manage Journal content (Table 1). For the *CHEST* staff team, I am thankful for important leadership from Nicki Augustyn, CAE, Senior Vice President of Publishing; Kiya Myers, MPS, Managing Editor; and Laura Riordan, MS, Director of Journals. Our partnership with Elsevier provides us access to experts in biomedical publication and allows us to remain on the forefront of publishing trends and technology. I have found a fast friend in Dr Hayley Gershengorn, Editor-in-Chief of *CHEST Critical Care*, as we share the experience of launching these new Journals. Last, I am indebted to Dr Peter Mazzone, Editor-in-Chief of *CHEST*, for his ongoing support and mentorship.

**Table 1:** *CHEST Pulmonary* Editorial Board

Editor-in-Chief	Matthew C Miles, MD, MEd, FCCP Atrium Health Wake Forest Baptist Medical Center
Associate Editors	
Asthma	Victor E. Ortega, MD, PhD Mayo Clinic
Chest Infections	Colin Swenson, MD Emory Healthcare
COPD	Auyon Ghosh, MD, MPH State University of New York–Upstate
Diffuse Lung Disease	Deborah Assayag, MD, MAS McGill University Health Centre
Education and Clinical Practice	Michelle Sharp, MD, MHS Johns Hopkins Medicine
Pulmonary Vascular	Mary Jo Farmer, MD, PhD Baystate Health
Sleep	Carolyn D'Ambrosio, MS, MD, FCCP Yale Medicine
Thoracic Oncology	Abhinav Agrawal, MD Northwell Health

By reading this brief announcement, you also join the *CHEST Pulmonary* team as our most key stakeholder: our audience. Thank you for starting this journey with us. I welcome your feedback and entreat your participation in the journal.

Information for Authors: https://www.chestpulmonary.org/infoforauthors

To submit your work: https://www.editorialmanager.com/chest-pulmonary/default2.aspx

## Financial/Nonfinancial Disclosures

None declared.
